# Trends in Unit Sales of Flavored and Menthol Electronic Cigarettes in the United States, 2012–2016

**DOI:** 10.5888/pcd15.170576

**Published:** 2018-08-23

**Authors:** Nicole M. Kuiper, Brett R. Loomis, Kyle T. Falvey, Doris G. Gammon, Brian A. King, Teresa W. Wang, Todd Rogers

**Affiliations:** 1 Office on Smoking and Health, National Center for Chronic Disease Prevention and Health Promotion, Centers for Disease Control and Prevention, Atlanta, Georgia; 2 Center for Health Policy Science and Tobacco Research, RTI International, Research Triangle Park, North Carolina; 3 Epidemic Intelligence Service, Centers for Disease Control and Prevention, Atlanta, Georgia

## Abstract

**Introduction:**

The use of flavored tobacco products, including electronic cigarettes (e-cigarettes), is common in the United States, and flavored products are particularly appealing to young people. The objective of this study was to describe national and state trends in flavored and menthol e-cigarette unit sales.

**Methods:**

We examined data on 4 types of e-cigarette products (rechargeables, disposables, prefilled cartridges, and e-liquid refills). We used Universal Product Code retail scanner data from 2 sources: 1) convenience stores and 2) all other outlets combined, including supermarkets, drug stores, mass merchandisers (including Walmart), dollar stores, club stores, and US Department of Defense commissaries. We aggregated data in 4-week periods for the 48 contiguous states and the District of Columbia for the 5-year period from 2012 through 2016. Data from vape shops and internet sales were not available. We used Joinpoint regression to assess trends.

**Results:**

From 2012 through 2016, flavored e-cigarette sales as a percentage of all e-cigarette sales increased nationally (from 2.4% to 19.8%) and in all but 4 states (North Dakota, South Dakota, Utah, and Vermont). Nationally, flavored disposable and prefilled cartridge sales increased. Menthol e-cigarette sales were stable nationally at 35% to 40%, while the percentage of menthol disposable, prefilled cartridge, and e-liquid refill sales decreased. By state, menthol e-cigarette sales increased in 2 states (Idaho and Nebraska) and decreased in 7 states. During 2015–2016, the percentage of flavored sales decreased in one state (Rhode Island) and increased in 29 states.

**Conclusion:**

These findings demonstrate that sales of flavored e-cigarette products have increased dramatically since 2012, with variations by product type and state. Continued monitoring of sales trends at all retail outlets can inform federal, state, and local efforts to address flavored tobacco product use, including e-cigarettes, in the United States.

## Introduction

The landscape of electronic cigarette (e-cigarette) use in the United States is evolving. Among adults, e-cigarette use increased from 2010 through 2013 and then plateaued, and use was primarily among current and former cigarette smokers (1,2). Current e-cigarette use among adolescents increased by 900% from 2011 through 2015 (3), before declining in 2016. E-cigarettes are the most commonly used tobacco product among adolescents (4).

The popularity of e-cigarettes is partly attributed to flavors (5–7). Flavors can make tobacco products particularly appealing to young people (5,8–11), and menthol flavors can mask tobacco’s harshness and promote initiation among young people (12). Most tobacco products used by young people are flavored (13,14); in 2014, 70% of middle and high school current tobacco users reported using flavored products (13). Flavored tobacco is a public health concern because flavored e-cigarette use is associated with higher levels of intention to initiate cigarette smoking among never-smoking adolescents, lower levels of intention to quit among adolescent smokers, and decreased perceptions of harm, compared with nonusers of e-cigarettes (15).

The health effects of inhaling aerosolized e-cigarette ingredients, including flavorants, are not completely understood. Levels of and exposure to toxicants can vary by liquid, user behavior, and the product’s heating element (5,16). Moreover, although some flavoring chemicals are approved for ingestion, they can have adverse health effects when inhaled (5,16). A recent comprehensive review indicated that e-cigarette aerosols can induce acute endothelial cell dysfunction and promote oxidative stress, increase diastolic blood pressure due to nicotine, and increase cough and wheeze in adolescent e-cigarette users (16).

Monitoring tobacco sales can complement self-reported surveillance methods to assess trends in tobacco use. Retail sales data can provide timely information for monitoring the consumption of tobacco products by venue, product type, and brand. Although studies have documented increases in e-cigarette sales (17–19), this is the first study to assess recent national and state trends in flavored and menthol e-cigarette product sales.

## Methods

We acquired data on estimates of US national and state sales of e-cigarettes and cigarettes from The Nielsen Company (www.nielsen.com/us/en.html). The Nielsen Company (Nielsen) collects data from a large, representative sample of retail outlets and applies a proprietary weighting method to project total sales in variously defined geographic areas. The data include Universal Product Code (UPC) sales from 2 distinct retail channels: convenience stores and all other outlets combined. Convenience stores include franchise, chain, and independent convenience stores that may or may not sell gasoline. The channel of all other outlets combined includes supermarkets, drug stores, mass merchandisers (including Walmart), dollar stores, club stores, and US Department of Defense commissaries. Data from vape shops, tobacco shops, or the internet were not commercially available.

Data were reported in 4-week aggregates, or approximately monthly. This analysis evaluated data from January 15, 2012, through January 7, 2017. Because we included only 7 days in 2017, we refer to the study period as 2012–2016. Combined data from convenience stores and all other outlets combined were available for the United States overall, the 48 contiguous states, and the District of Columbia. Because Nielsen did not provide data for Alaska or Hawaii, these states were not included in the national estimates.

### Measures

Data from Nielsen consisted of sales metrics, such as dollars and units, and product characteristics for each UPC, including brand name, product type, flavor, and number of items per unit (eg, a 5-pack of disposable e-cigarettes). Each e-cigarette UPC was assigned to one of 4 categories: 1) rechargeable e-cigarettes and e-cigarette starter kits (rechargeables), 2) disposable e-cigarettes (disposables), 3) cartridges prefilled with e-liquid (prefilled cartridges), and 4) bottles of e-liquid refills (e-liquids). Rechargeables are packaged with at least one rechargeable battery, a refill of e-liquid, and a battery charger. Disposables have a nonrechargeable battery and a nonrefillable e-liquid reservoir. Prefilled cartridges can be disposable or refillable. Bottles of e-liquids are used to refill depleted cartridges or tank systems.

All e-cigarette products were further sorted into one of 3 flavor categories: flavored (eg, fruit, spice, vanilla, chocolate, coffee, alcohol); menthol (eg, wintergreen, ice mint); and regular (eg, original, regular tobacco). We used information from the brand’s website or online retailers to categorize abstract or ambiguous flavor descriptions (eg, Purple Haze). We coded UPCs with both flavor and menthol characterizations (eg, watermelon mint) as flavored. UPCs corresponding to multiple items of differing flavors (eg, a starter kit that includes one menthol-flavored cartridge and one regular-flavored cartridge) could not be sorted reliably into one of the 3 flavor categories, so we excluded these from analysis. UPCs of this type accounted for less than 1% of total unit sales. One researcher (K.T.F.) prepared the data set and conducted the initial analysis, and a second researcher (D.G.G.) reviewed the analysis.

### Analysis

Unit sales were standardized by the number of items per UPC to represent the most commonly purchased quantities in each product category. After standardization, one standardized unit equaled one rechargeable, one disposable, one package of 5 prefilled cartridges, or one bottle of e-liquid. We determined the total number of unit sales of e-cigarettes, by flavoring type (flavored, menthol, and regular), in 4-week aggregates. For all e-cigarettes and for each of the 4 product categories, we counted the number of unique UPCs during six 4-week periods; we then calculated the percentage of flavored products and menthol products in each category. We calculated unit sales of flavored and menthol products as a percentage of sales for each of 4 types of e-cigarette products and all e-cigarettes (eg, menthol unit sales as a percentage of unit sales of disposables) in 4-week aggregates. For all e-cigarettes and for each of the 4 product categories, we determined the total number of unit sales, the percentage of flavored unit sales, the annual monthly percentage change (AMPC) in flavored unit sales, the percentage of menthol unit sales, and the AMPC for menthol unit sales; we determined these values by year for the study period. As a comparison, we determined the total unit sales of combustible cigarettes, the percentage of menthol unit sales, and the AMPC for menthol units. Finally, for states, we calculated the percentages of flavored and menthol e-cigarette unit sales from 2012 through 2016 and for 2015–2016 and the AMPC for each year.

We used Joinpoint software version 4.5.0.1 (Information Management Systems, Inc) to test for significance of discrete changes in trends in the percentages of sales (log-transformed), by flavor category, during the study period. Use of a log-linear model allows for analysis of percentage change in each outcome over time. Joinpoint is a segmented regression software program developed and disseminated by the US National Cancer Institute that utilizes a data-driven method to identify structural breaks, or “joinpoints,” in trends and produce easily interpretable post-regression results for average AMPCs, accounting for the length between breaks. For each e-cigarette product category, the AMPCs, which measure the relative change during the time period, were assessed by year and over the full study period. The independent variable in each joinpoint regression was a continuous time trend that corresponded to the reporting periods (1 to 65). National and state trends were assessed, where applicable, under the null hypothesis that AMPCs were neither increasing nor decreasing (*P* < .05). The optimal number of joinpoints for each trend was assessed by using the Bayesian Information Criterion, and all models controlled for serial correlation.

## Results

### National sales data

#### Total sales

**Figure 1:**
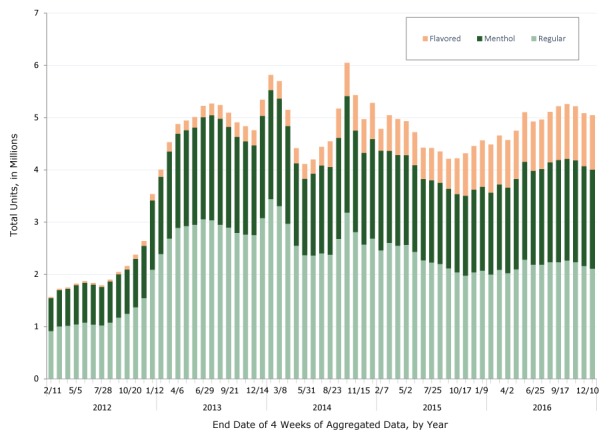
Total units of e-cigarette sales (in millions), by flavoring type, United States, 2012–2016. Data are reported in 4-week aggregates, with aggregates ending on the dates indicated. Data source: The Nielsen Company. **Table:** 

**Figure 2:**
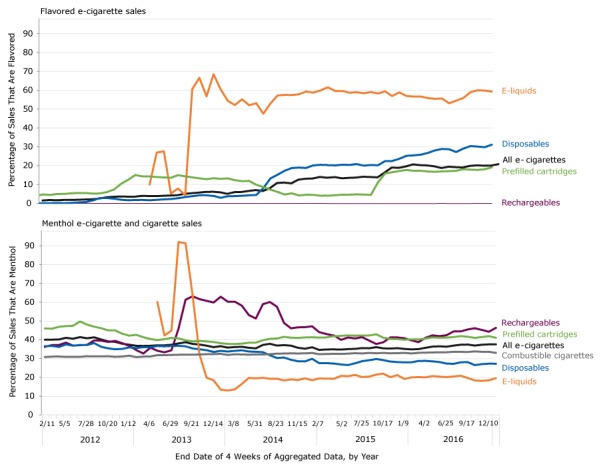
Unit sales of flavored and menthol products as a percentage of sales for each of 4 types of e-cigarette products (rechargeables, disposables, prefilled cartridges, and e-liquids) and all e-cigarettes, United States, 2012–2016. Data are reported in 4-week aggregates, with aggregates ending on the dates indicated. Flavored and menthol e-liquids sales began at the 4-week period ending May 4, 2013. Data source: The Nielsen Company. **Table:** 

E-cigarette sales increased by 150%, from 1.6 million units in January 2012 to 4 million units in February 2013 (Figure 1). From then until the end of 2016, sales remained at 4 to 6 million units. The percentage of flavored sales increased from 1.5% of the 1.6 million units sold in January 2012 to 20.7% of the 5.0 million units sold in December 2016 (Figure 2). The percentage of menthol sales was stable at just under 40% during the study period, with decreases during 2013–2015 and increases during 2016.

Year/End Date of 4 Weeks of Aggregated Data	Flavored	Menthol	Regular
**2012**			
2/11	0.023218	0.627003	0.917202296
3/10	0.029810	0.691038	1.004283618
4/7	0.028174	0.702246	1.018259326
5/5	0.032600	0.747519	1.042826642
6/2	0.033373	0.757946	1.079374672
6/30	0.034618	0.759271	1.041052311
7/28	0.036663	0.730313	1.025010005
8/25	0.040191	0.784254	1.076528601
9/22	0.051799	0.820971	1.173341683
10/20	0.069790	0.845008	1.24610567
11/17	0.082711	0.923271	1.370236679
12/15	0.095081	0.999271	1.545142714
1/12/2013	0.124093	1.320573	2.093429212
**2013**			
2/9	0.140559	1.473389	2.391570495
3/9	0.179646	1.662699	2.687320888
4/6	0.188698	1.794275	2.895699906
5/4	0.187322	1.832041	2.926362636
6/1	0.201662	1.855797	2.953385640
6/29	0.218099	1.950232	3.056014268
7/27	0.227526	2.005116	3.038235142
8/24	0.263420	2.027189	2.950871305
9/21	0.274263	1.925129	2.896626087
10/19	0.278323	1.837122	2.793897979
11/16	0.293841	1.781790	2.762040859
12/14	0.291359	1.714566	2.752742571
1/11	0.312321	1.952568	3.079075464
**2014**			
2/8	0.293349	2.083041	3.442486783
3/8	0.336298	2.056144	3.307353690
4/5	0.310120	1.865806	2.971462223
5/3	0.290458	1.575655	2.549008086
5/31	0.288519	1.456991	2.368614392
6/28	0.273506	1.563459	2.361264443
7/26	0.358427	1.679382	2.402648834
8/23	0.491512	1.675955	2.378292658
9/20	0.568431	1.921186	2.685060207
10/18	0.640599	2.227296	3.182321125
11/15	0.680826	1.939113	2.811662204
12/13	0.647783	1.750016	2.572831207
1/10/2015	0.694899	1.902111	2.687018836
**2015**			
2/7	0.670059	1.654252	2.461748398
3/7	0.685748	1.758624	2.603786971
4/4	0.688567	1.733114	2.550768811
5/2	0.650289	1.714433	2.566036646
5/30	0.634152	1.652124	2.434724847
6/27	0.602143	1.556255	2.268506025
7/25	0.619875	1.570013	2.228358228
8/22	0.603490	1.542535	2.207593838
9/19	0.576550	1.519386	2.116696347
10/17	0.685608	1.494048	2.041021508
11/14	0.815051	1.524683	1.977724943
12/12	0.836162	1.581678	2.038924235
1/9/2016	0.890760	1.601680	2.072417962
**2016**			
2/6	0.923495	1.564532	1.999259463
3/5	0.940893	1.630360	2.086987273
4/2	0.916145	1.631938	2.025089904
4/30	0.928321	1.726583	2.095914468
5/28	0.953164	1.862395	2.288982348
6/25	0.951490	1.786446	2.187446171
7/23	0.948608	1.829414	2.185020535
8/20	0.967632	1.907034	2.234566395
9/17	1.032938	1.953210	2.233575448
10/15	1.056684	1.940104	2.265064284
11/12	1.037423	1.947240	2.232956149
12/10	1.015580	1.906919	2.161599276
1/7/2017	1.044052	1.894045	2.108576583

Year/End Date	Percentage Flavored					Percentage Menthol					
	All E-Cigarettes	Rechargeables	Disposables	E-liquids	Cartridge Refills	All E-Cigarettes	Rechargeables	Disposables	E-liquids	Cartridge Refills	Combustible Cigarettes
**2012**											
2/11	1.5	0	0	—	3.9	40.0	36.2	36.6	—	46.0	30.8
3/10	1.7	0	0.1	—	4.7	40.1	37.1	36.7	—	45.8	31.0
4/7	1.6	0	0.1	—	4.4	40.2	37.2	36.2	—	46.9	31.1
5/5	1.8	0	0.2	—	4.9	41.0	38.5	37.5	—	47.2	30.9
6/2	1.8	0	0.1	—	5.0	40.5	36.9	36.8	—	47.4	30.9
6/30	1.9	0	0.2	—	5.3	41.4	37.1	37.2	—	49.7	30.9
7/28	2.0	0	0.5	—	5.5	40.8	37.3	37.2	—	48.0	31.2
8/25	2.1	0	0.7	—	5.4	41.3	39.6	38.2	—	46.9	31.2
9/22	2.5	0	1.5	—	5.2	40.1	39.5	36.2	—	46.1	31.2
10/20	3.2	0	2.8	—	5.2	39.1	38.8	35.4	—	44.9	31.2
11/17	3.5	0	2.8	—	6.0	38.9	39.4	34.9	—	45.0	30.9
12/15	3.6	0	2.4	—	7.4	37.9	38.2	35.1	—	43.2	31.1
1/12	3.5	0	1.9	—	10.4	37.3	36.7	35.9	—	42.2	31.5
**2013**											
2/9	3.5	0	1.6	—	12.5	36.8	34.5	35.6	—	42.6	30.7
3/9	4.0	0	1.8	—	15.0	36.7	32.7	36.1	—	41.3	31.0
4/6	3.9	0	1.8	—	14.3	36.8	35.8	36.0	—	40.4	31.1
5/4	3.8	0	1.6	10.0	14.3	37.0	34.1	36.6	60.0	39.8	31.8
6/1	4.0	0	1.9	26.9	13.9	37.0	33.3	36.5	42.3	40.3	31.8
6/29	4.2	0	2.1	27.6	13.7	37.3	34.4	36.6	44.8	41.0	31.9
7/27	4.3	0	2.3	5.2	13.5	38.0	45.8	36.7	91.9	40.7	32.0
8/24	5.0	0	2.7	7.8	15.0	38.7	61.3	36.5	91.4	39.7	32.1
9/21	5.4	0	3.3	4.3	14.3	37.8	63.1	35.4	63.6	39.1	32.0
10/19	5.7	0	3.7	60.6	13.8	37.4	61.6	34.9	32.0	39.3	32.2
11/16	6.1	0	4.3	66.6	13.3	36.8	60.7	34.4	19.8	39.1	32.3
12/14	6.1	0	4.3	56.7	12.8	36.0	59.8	33.5	18.4	38.7	32.4
1/11	5.8	0	3.9	68.5	13.3	36.5	62.9	34.1	13.4	38.2	32.5
**2014**											
2/8	5.0	0	2.9	60.4	13.0	35.8	60.3	33.8	13.0	37.8	31.9
3/8	5.9	0	3.7	54.3	13.2	36.1	60.2	34.1	13.6	37.7	32.3
4/5	6.0	0	3.8	52.2	12.3	36.2	58.1	34.3	16.5	37.9	32.1
5/3	6.6	0	4.0	55.2	11.7	35.7	53.2	33.7	19.7	38.4	32.0
5/31	7.0	0	4.2	52.0	11.9	35.4	51.3	33.6	19.4	38.5	32.1
6/28	6.5	0	4.4	53.1	9.9	37.2	58.9	33.4	19.7	39.7	32.2
7/26	8.1	0	8.1	47.6	8.6	37.8	60.0	31.7	19.3	40.5	32.3
8/23	10.8	0	13.2	52.9	7.3	36.9	57.5	30.3	19.2	40.7	32.6
9/20	11.0	0	15.2	57.2	6.1	37.1	48.9	30.5	18.3	41.5	32.6
10/18	10.6	0	17.3	57.5	4.6	36.8	46.0	29.3	18.9	41.1	32.7
11/15	12.5	0	18.7	57.5	5.3	35.7	46.6	28.6	18.6	41.0	32.7
12/13	13.0	0	19.0	57.8	4.1	35.2	46.7	28.4	19.4	41.2	32.7
1/10/2015	13.2	0	18.8	59.3	4.5	36.0	47.2	29.9	18.5	41.3	33.0
**2015**											
2/7	14.0	0	20.1	58.7	4.5	34.6	44.0	27.5	19.4	41.1	32.3
3/7	13.6	0	20.4	59.8	4.0	34.8	43.1	27.6	19.3	41.2	32.4
4/4	13.8	0	20.3	61.5	4.2	34.9	42.3	27.3	19.2	41.9	32.5
5/2	13.2	0	20.2	59.6	4.4	34.8	39.9	26.8	20.8	42.1	32.4
5/30	13.4	0	20.6	59.7	4.7	35.0	41.1	26.6	20.6	42.3	32.6
6/27	13.6	0	20.5	58.8	4.7	35.2	40.7	27.5	21.3	42.2	32.8
7/25	14.0	0	20.9	59.0	4.8	35.5	41.4	28.6	20.2	42.2	32.7
8/22	13.9	0	20.1	58.5	4.7	35.4	39.5	29.1	20.4	42.3	33.0
9/19	13.7	0	20.4	59.1	4.5	36.1	37.7	29.7	21.4	42.9	32.8
10/17	16.2	0	20.3	58.3	11.2	35.4	38.6	29.2	22.0	41.1	33.0
11/14	18.9	0	22.5	59.4	15.9	35.3	41.2	28.4	20.1	40.4	32.8
12/12	18.8	0	22.5	57.0	16.6	35.5	41.2	28.1	21.2	40.3	32.8
1/9/2016	19.5	0	23.7	58.9	17.2	35.1	40.8	28.0	19.1	40.0	33.0
**2016**											
2/6	20.6	0	25.3	57.1	17.7	34.9	39.7	28.0	20.0	40.2	32.7
3/5	20.2	0	25.5	56.7	17.4	35.0	38.7	28.7	20.1	40.2	33.0
4/2	20.0	0	25.9	56.7	17.4	35.7	41.0	28.8	20.0	40.6	33.1
4/30	19.5	0	26.8	55.9	17.0	36.3	42.3	28.5	20.7	41.2	33.2
5/28	18.7	0	28.2	55.5	16.8	36.5	41.9	28.1	20.4	41.0	33.3
6/25	19.3	0.1	28.9	55.7	17.0	36.3	42.4	27.3	20.1	41.1	33.3
7/23	19.1	0	28.8	53.1	17.2	36.9	44.4	27.2	20.5	41.4	33.6
8/20	18.9	0	27.3	54.6	17.3	37.3	44.4	28.0	20.9	41.9	33.5
9/17	19.8	0	29.1	56.0	18.2	37.4	45.4	28.0	19.7	41.5	33.5
10/15	20.1	0	30.5	59.0	18.0	36.9	46.1	26.5	18.4	41.0	33.8
11/12	19.9	0.1	30.2	60.1	17.8	37.3	45.2	27.0	18.2	41.4	33.6
12/10	20.0	0.1	29.9	59.9	18.2	37.5	44.2	27.3	18.4	41.9	33.6
1/7/2017	20.7	0.1	31.2	59.3	19.2	37.5	46.3	27.2	19.6	41.0	33.0

#### Product proliferation

From 2012 through 2016, the increase in total e-cigarette unit sales corresponded to an increase in the number of unique e-cigarette products on the market (Table 1). The number of unique e-cigarette products increased 190%, from 195 products in 2012 to 565 in 2016. For the study period, the percentage of unique e-cigarette products that were flavored increased from 11% to 44%. In contrast, the percentage that were menthol decreased from 37% to 24%. By late 2016, 78% of e-liquid refill products were flavored and 11% were menthol, surpassing the combined percentage of flavored and menthol products for disposables (67%), prefilled cartridges (59%), and rechargeables (40%).

**Table 1:** Number of Unique E-Cigarette Products on the US Market, by Universal Product Code, and the Percentage of Products That Were Flavored or Menthol, United States, 2012–2016^a^

Product	Measure	4-Week Period Ending on . . .					
		Feb 11, 2012^b^	Feb 9, 2013	Feb 8, 2014	Feb 7, 2015	Feb 6, 2016	Jan 7, 2017^c^
All e-cigarettes	No. of unique UPCs	195	335	473	608	594	565
	Flavored, %^d^	11	20	29	42	43	44
	Menthol, %^d^	37	33	29	26	25	24
Rechargeables	No. of unique UPCs	43	59	64	62	61	63
	Flavored, %^d^	NA	NA	NA	NA	NA	3
	Menthol, %^d^	40	41	41	42	41	37
Disposables	No. of unique UPCs	80	168	256	256	220	184
	Flavored, %^d^	11	28	38	43	43	42
	Menthol, %^d^	33	27	24	24	24	25
Prefilled cartridges	No. of unique UPCs	72	108	124	139	147	157
	Flavored, %^d^	18	19	19	22	20	26
	Menthol, %^d^	42	39	36	37	36	33
E-liquids^e^	No. of unique UPCs	NA	3	29	151	166	161
	Flavored, %^d^	NA	33	59	77	78	78
	Menthol, %^d^	NA	33	21	12	12	11

Abbreviation: UPC, Universal Product Code.

a The number of unique products was determined by UPCs. Data source: The Nielsen Company. Data span January 15, 2012, through January 7, 2017. Sales are combined sales from convenience stores and all other outlets combined. Excludes Alaska and Hawaii.

b The first 4-week period in the data.

c The last 4-week period in the data.

d Percentage was calculated as the number of unique UPCs for flavored products (or menthol products) divided by the number of unique UPCs in the product category. For example, of the number of unique UPCs for all e-cigarettes for the 4-week period ending February 11, 2012, 11% were flavored and 37% were menthol. The remaining proportion in each product category is nonflavored, nonmenthol tobacco (ie, regular tobacco).

e Flavored and menthol e-liquid refill sales began at the 4-week period ending May 4, 2013.

#### Flavored and menthol sales, by product category

We found significant increases and decreases in the sales of several products as a percentage of total sales during the study period (Figure 2 and Table 2). The sales of flavored and menthol e-liquids fluctuated during the first 6 months of their introduction into the e-cigarette market (Figure 2). The sales of flavored disposables and flavored prefilled cartridges increased from 2012 through 2016, although percentages varied year to year. In 2016, nearly 20% of all e-cigarettes sold were flavored. In 2016, the highest percentage of flavored unit sales were for e-liquids (56.9%), followed by disposables (28.3%) and prefilled cartridges (17.6%). Sales percentages of menthol disposables, prefilled cartridges, and e-liquids decreased during the study period, although percentages varied from year to year. In 2016, just over one-third (36.6%) of all e-cigarette sales were menthol; the highest percentage of menthol unit sales were for rechargeables (43.2%), followed by prefilled cartridges (41.1%) and disposables (27.7%).

**Table 2:** Number of Unit Sales^a^ of Flavored and Menthol E-Cigarettes and Annual Monthly Percentage Change (AMPC)^b^ in Unit Sales, United States, 2012–2016^c^

Product	Measure	2012	2013	2014	2015	2016	2012–2016
All e-cigarettes	Total no. of units^a^	27.0	64.1	65.3	59.4	64.4	280.2
	Flavored, %^d^	2.4	4.8	8.9	15.1	19.8	10.2
	AMPC^b^ for flavored units	7.9^e^	3.8^e^	6.9^e^	1.9^e^	1.9^e^	4.4^e^
	Menthol, %^d^	39.9	37.2	36.3	35.2	36.6	37.0
	AMPC^b^ for menthol units	−0.6	−0.2^e^	−0.2^e^	−0.2^e^	0.7^e^	−0.1
Rechargeables	Total no. of units^a^	4.1	4.0	7.9	7.8	10.7	34.5
	Flavored, %^d^	0	0	0	0	0	0
	AMPC^b^ for flavored units	NA	NA	NA	NA	NA	NA
	Menthol, %^d^	37.9	47.7	53.5	40.9	43.2	44.6
	AMPC^b^ for menthol units	−0.6	4.6^e^	−2.2^e^	−1.1^e^	1.2^e^	0.3
Disposables	Total no. of units^a^	14.2	47.3	36.6	21.1	18.6	137.8
	Flavored, %^d^	1.0	2.7	10.3	21.0	28.3	12.6
	AMPC^b^ for flavored units	43.3^e^	6.2	14.3^e^	1.8^e^	1.8^e^	11.7^e^
	Menthol, %^d^	36.4	35.6	31.7	28.0	27.7	31.9
	AMPC^b^ for menthol units	−0.3^e^	−0.3^e^	−1.6^e^	0.2	−0.5^e^	−0.5^e^
Prefilled cartridges	Total no. of units^a^	8.7	12.8	18.9	26.4	31.9	98.7
	Flavored, %^d^	5.6	13.8	8.7	7.8	17.6	10.7
	AMPC^b^ for flavored units	8.5^e^	1.9^e^	−8.2^e^	11.6^e^	0.7	2.6^e^
	Menthol, %^d^	46.1	40.0	39.8	41.5	41.1	41.7
	AMPC^b^ for menthol units	−0.5^e^	−0.8^e^	0.7^e^	0	0	−0.2^e^
E-liquids	Total no. of units^a^	0	0.03	1.9	4.1	3.2	9.3
	Flavored, %^d^	0	33.4	55.2	59.1	56.9	52.2
	AMPC^b^ for flavored units	NA	11.0	−0.1	−0.1	−0.1	1.9
	Menthol, %^e^^,^ f	0	47.8	18.0	20.4	19.8	25.2
	AMPC^b^ for menthol units	NA	−14.1^e^	3.6^e^	0	0	−1.7^e^
Combustible cigarettes	Total no. of units^a^	11,596.8	11,350.5	11,067.0	11,021.7	10,717.5	55,753.5
	Menthol, %	31.1	31.8	32.4	32.7	33.3	32.3
	AMPC^b^ for menthol units	0	0.3^e^	0.1^e^	0.1^e^	0.1	0.1^e^

a Units are reported in millions.

b AMPC is the relative change within each 4-week period.

c Data source: The Nielsen Company. Data span January 15, 2012, through January 7, 2017. Sales represent combined sales from convenience stores and all other outlets combined. Excludes Alaska and Hawaii.

d Percentage was calculated as the number of sales units of flavored products (or menthol products) divided by the total number of sales units in the product category. For example, of the total number of sales units for all e-cigarettes in 2012, 2.4% were flavored and 39.9% were menthol. The remaining proportion in each product category is nonflavored, nonmenthol tobacco (ie, regular tobacco).

e AMPC was significantly different from zero (*P* < .05).

f 2013 sales of flavored and menthol liquid e-cigarettes began on April 7.

#### Flavored and menthol sales, by year

Overall, unit sales of flavored e-cigarettes as a percentage of unit sales of all e-cigarettes increased, from 2.4% in 2012 to 19.8% in 2016 (Table 2). Averaged across the study period, more than half (52.2%) of e-liquids sales were flavored, and 3.3% (9.3 million of 280.2 million) of all e-cigarette sales were sales of e-liquids.

From 2012 through 2016, the unit sales of flavored products as a percentage of unit sales for all disposables increased from 1.0% to 28.3% and for all prefilled cartridges from 5.6% to 17.6%. In contrast, despite year-to-year variations, the unit sales of menthol e-cigarette as a percentage of all unit sales of e-cigarettes was stable, from 39.9% in 2012 to 36.6% in 2016. The unit sales of menthol products as a percentage of unit sales for all disposables declined (36.4% in 2012 to 27.7% in 2016), for all prefilled cartridges (46.1% in 2012 to 41.1% in 2016), and for all e-liquids (47.8% in 2013 to 19.8% in 2016), despite year-to-year variations for all product types. For combustible cigarettes, the percentage of unit sales of menthol products increased, from 31.1% in 2012 to 33.3% in 2016.

### State sales data

#### Flavored and menthol e-cigarette sales, by year

From 2012 through 2016, by state, the unit sales of flavored e-cigarettes as a percentage of all e-cigarettes was highest in New Mexico (18.5%), and the unit sales of menthol e-cigarettes as a percentage of all e-cigarettes was highest in Rhode Island (46.0%) (Table 3). Percentages of flavored e-cigarette sales increased in 45 states from 2012 through 2016 and was unchanged in 4 states (North Dakota, South Dakota, Utah, and Vermont). Percentages of menthol e-cigarette sales decreased in 7 states (California, District of Columbia, Florida, Minnesota, New Jersey, New York, and Pennsylvania) and increased in 2 states (Idaho and Nebraska) during the study period.

**Table 3:** Trends^a^ in Unit Sales of Flavored and Menthol E-Cigarettes as a Percentage of All E-Cigarette Unit Sales, by Region and State, United States, 2012–2016^b^

US Census Region/State	Percentage of Flavored		Percentage of Menthol	
	2012–2016	2015–2016	2012–2016	2015–2016
**Northeast**				
Connecticut	7.6^c^	12.7^c^	37.0	32.4
Massachusetts	7.9^c^	13.6	34.0	33.5^c^
Maine	11.4^c^	20.2^c^	26.3	26.4^c^
New Hampshire	11.5^c^	21.0^c^	30.6	30.0^c^
New Jersey	5.1^c^	8.8^c^	45.6^d^	44.6
New York	5.2^c^	8.7^c^	42.0^d^	39.3
Pennsylvania	9.3^c^	15.0^c^	43.0^d^	43.3^c^
Rhode Island	3.9^c^	6.0^d^	46.0	44.4
Vermont	5.1	9.1^c^	31.9	29.9
**South**				
Alabama	9.9^c^^,^ e	16.2^c^	37.0	37.2^c^
Arkansas	10.1^c^	19.3^c^	31.0	26.9
Delaware	10.2^c^	20.1	37.6	35.8
District of Columbia	12.1^c^^,^ e	21.8^c^	40.7^d^	38.4
Florida	10.9^c^	19.5^c^	35.7^d^	32.9
Georgia	11.0^c^	18.2^c^	38.2	38.5
Kentucky	9.2^c^	15.6	29.9	29.4^c^
Louisiana	9.3^c^	16.1^c^	39.0	39.7
Maryland	9.8^c^	19.4	41.9	39.6^c^
Mississippi	9.8^c^^,^ e	16.8^c^	40.1	37.7
North Carolina	11.4^c^	17.3^c^	39.2	36.7
Oklahoma	12.1^c^	21.4^c^	27.9	27.4
South Carolina	12.6^c^	18.9^c^	40.0	38.9
Tennessee	10.3^c^	16.8	33.5	33.5
Texas	8.4^c^	15.7^c^	37.3	36.0
Virginia	10.7^c^	20.2	38.1	36.4^c^
West Virginia	13.5^c^	20.7	31.3	30.6
**Midwest**				
Iowa	12.8^c^	22.7	30.5	28.8
Illinois	12.7^c^	22.3^c^	38.9	36.7^c^
Indiana	10.0^c^	18.2	34.9	34.8
Kansas	12.2^c^	20.9^c^	33.2	32.2^c^
Michigan	8.5^c^	13.9	39.7	40.1
Minnesota	12.5^c^^,^ e	25.7^c^	31.1^d^	26.3^d^
Missouri	10.9^c^	20.5^c^	33.9	32.5^c^
North Dakota	9.9	13.9^c^	26.1	15.0
Nebraska	12.7^c^	22.1^c^	32.3^c^	34.2^c^
Ohio	9.0^c^	15.3	35.2	35.2^c^
South Dakota	11.4	18.1	37.1	36.8
Wisconsin	12.1^c^	21.2	39.9	37.7
**West**				
Arizona	18.1^c^	28.0	30.6	27.8^c^
California	13.5^c^	22.0^c^	33.9^d^	33.3^c^
Colorado	14.1^c^	20.3	32.2	33.2
Idaho	14.3^c^	26.8	31.4^c^	30.2
Montana	12.6^c^	22.7^c^	30.5	27.6
New Mexico	18.5^c^^,^ e	30.8	29.6	27.8^c^
Nevada	13.1^c^	24.3^c^	31.5	29.7^c^
Oregon	11.5^c^	20.0	27.7	28.1^c^
Utah	17.6	26.5	38.5	36.5
Washington	11.6^c^	20.2^c^	35.4	32.9
Wyoming	13.0^c^	21.3^c^	28.5	26.3

Abbreviation: AMPC, average monthly percentage change.

a Trends were measured by determining AMPCs; AMPC is the relative change within each 4-week period.

b Data source: The Nielsen Company. Data span January 15, 2012, through January 7, 2017. Sales are combined sales from convenience stores and all other outlets combined. Excludes Alaska and Hawaii.

c Positive AMPC, significantly different from zero at α = .05.

d Negative AMPC, significantly different from zero at α = .05.

e Trend begins July 29, 2012, because of gaps in earlier data on flavored or menthol sales.

During 2015–2016, by state, the unit sales of flavored e-cigarettes as a percentage of unit sales of all e-cigarettes was highest in New Mexico (30.8%), and the unit sales of menthol e-cigarettes as a percentage of all e-cigarettes was highest in New Jersey (44.6%). The percentage of flavored sales decreased in one state (Rhode Island) and increased in 29 states during 2015–2016. During the same period, percentages of menthol e-cigarette sales decreased in one state (Minnesota) and increased in 18 states.

## Discussion

From 2012 through 2016, the number of unique e-cigarette types available in traditional retail outlets in the United States increased 190%, and the percentage of those that were flavored increased from 11% to 44%. In 2014, at least 466 unique brands of e-cigarettes and 7,764 unique flavors were available on the market (7). This product proliferation from 2012 through 2016 corresponded to an increase in sales. From 2012 through 2016, flavored e-cigarette sales increased significantly. More than half (56.3%) of total e-cigarette sales in 2016 were flavored or menthol products, and sales varied significantly by product type. These findings are consistent with those of previous studies that documented the recent widespread growth in US e-cigarette sales (17–19) and the prominent use of flavored e-cigarettes among US adolescents and adults (5,6,13–15). Similar to sales of e-cigarettes, sales of many other flavored and menthol combustible and noncombustible tobacco products also increased from 2011 through 2015 (20). Thus, continued monitoring of the diversity of flavored tobacco products available on the US market, including combustible, noncombustible, and electronic tobacco products, is critical for informing comprehensive tobacco prevention and control strategies in the United States.

Evidence-based population-level strategies can and have been implemented to minimize the potential adverse effects of e-cigarette use among vulnerable populations, such as adolescents, and could also maximize the opportunity to realize potential benefits of e-cigarettes among adult smokers who use the products to switch completely from combustible cigarettes (5,21). Federal law prohibits e-cigarette sales to people aged under 18 years. It also prohibits the distribution of free samples of e-cigarettes and vending machine sales of e-cigarettes, except in adult-only facilities. Moreover, 5 states, the District of Columbia, and more than 200 communities have increased the minimum age of sale for e-cigarettes to 21 years (21). Establishing and enforcing 21 years as the minimum age for all tobacco products, including e-cigarettes, as well as other retail-level policies can help reduce the potential for access to and use of these products among adolescents and young adults yet still allow e-cigarettes to be purchased by adult smokers who might use these products to quit smoking completely (5,21). Because adolescents are more likely to obtain e-cigarettes from friends, family, or peers than to purchase them, increasing the minimum age could minimize peer supply; moreover, acquisition through peers and family members suggests the importance of not only enforcing existing policies but also considering noncommercial sources of products for adolescents when designing and implementing tobacco prevention efforts (22). Other effective strategies to deter access to e-cigarettes among adolescents include eliminating self-service displays and implementing other retail-level policies (5,21). Similarly, incorporating e-cigarettes into indoor smoke-free laws can also prevent involuntary exposure among vulnerable populations and reduce the potential for the renormalization of tobacco product use, while still allowing adults who may wish to use e-cigarettes to attempt to quit smoking conventional cigarettes to do so in an outside area that does not involuntarily expose bystanders to e-cigarette aerosol (5,21).

The dramatic growth in the percentage of flavored e-cigarette sales during the study period contrasted with patterns of menthol sales, which were stable and substantial, despite year-to-year changes in sales and product types. The high percentage of menthol sales (averaging 37%) and increases in flavored sales are consistent with surveillance findings that demonstrate a consumer preference for flavored and menthol products (5,6,13–15). The percentage of menthol e-cigarette sales are generally consistent with those of menthol cigarettes, which make up about one-third of total cigarette sales (23). Among current tobacco users in the Population Assessment of Tobacco and Health (PATH) study, 80% of adolescents aged 12 to 17 years, 73% of young adults aged 18 to 24 years, and 63% of adults aged 25 to 29 years were current users of a flavored tobacco product. Of those 3 age groups, 16%, 33%, and 49%, respectively, of use was exclusively menthol cigarette smoking (14). Combined with the previous surveillance data, our findings highlight the importance of tobacco control strategies to address flavors that include mentholated tobacco products (12). Research is also warranted to assess perceived health consequences, product appeal to young people (24,25), and cessation behaviors among users of other flavored and menthol tobacco products (12,15,26).

The high percentages of — and observed differences in — trends of flavored and menthol sales across states highlight the value of evidence-based tobacco prevention and control strategies at state or local levels. In the United States, cigarettes with characterizing flavors, excluding tobacco and menthol, are prohibited under the 2009 Family Smoking Prevention and Tobacco Control Act; in May 2016, the US Food and Drug Administration (FDA) issued a final regulation extending its authority to regulate all tobacco products, including e-cigarettes (27). However, characterizing flavors are not currently prohibited in noncigarette products (5). Although regulation of tobacco product constituents is reserved for the FDA, states and municipalities are not preempted from enacting policies restricting sales of entire classes of products. In recent years, several jurisdictions in California, Illinois, Massachusetts, Minnesota, New York, and Rhode Island restricted the sale of flavored tobacco products (28). For example, in 2012, Providence, Rhode Island, prohibited flavored e-cigarette sales, excluding menthol, except in certain bars primarily devoted to the serving of tobacco products (28). In our study, Rhode Island was the only state with a significant decline in flavored e-cigarette sales during 2015–2016, but it had the second highest rate of menthol sales (44.4%). Additionally, 5 communities in California enacted jurisdiction-wide flavor bans, including menthol, with no retailers exempted, while other communities in California, Illinois, and Minneapolis passed similar but less stringent policies (eg, exemptions for certain retailers, prohibition in buffer zones) (28). Rigorously evaluating the implementation and impact of such policies will be critical to assess the effect of restricting flavored tobacco products on tobacco-related behaviors (29).

Our study has at least 2 limitations. First, sales from vape shops, tobacco shops, the Internet, and other nontracked channels were not available. These channels are projected to account for as much as $3.75 billion (62%) of the $5.5 billion e-cigarette market in 2018 (30). Thus, scanner data may underestimate the percentage of flavored e-cigarette sales. However, the findings are representative of the traditional tobacco retail market accessible to young people and consumers who do not visit vape shops or buy online. Second, the e-cigarette market is dynamic, and sales estimates can quickly become outdated because of product diversity. Ongoing monitoring of retail e-cigarette sales is important to understand how product availability and the changing tobacco regulatory environment affect consumer preferences.

This study demonstrates the increasing availability and diversity of flavored e-cigarettes in retail locations accessible to adolescents in the US market. To better understand the net impact of e-cigarettes on public health, including potential harms and benefits, it is crucial to understand what products are being purchased to develop appropriate measures to assess marketing, sales, perceptions, and use of these products (5). Therefore, data sources for measuring sales in nontracked channels, such as vape shops and the internet, are becoming increasingly important as the market continues to diversify. In the interim, the US Surgeon General has noted that sufficient evidence exists to justify public health strategies to minimize the established risks on population health, particularly for adolescents and young adults. These strategies could include educational initiatives, incorporating e-cigarettes into smoke-free policies, advertising, promotion and self-service display restrictions, restricting minors’ access to tobacco, flavor restrictions, and other retail-level polices (5). Sustained implementation of these strategies at local, state, tribal, and territorial levels — in coordination with regulation of the manufacturing, distribution, and marketing of all tobacco products — could help minimize population-level health risks, particularly for young people, while still affording the opportunity for a potential benefit to adult smokers who use e-cigarettes to quit smoking completely (5).
